# Study of the Stability of Palletized Cargo by Dynamic Test Method Performed on Laboratory Test Bench

**DOI:** 10.3390/s21155129

**Published:** 2021-07-29

**Authors:** Sławomir Tkaczyk, Mikołaj Drozd, Łukasz Kędzierski, Krzysztof Santarek

**Affiliations:** 1 Faculty of Transport, Warsaw University of Technology, Koszykowa 75, 00-662 Warsaw, Poland; 2 Ergis Load Stability Academy, Zwierzyniecka 12, 55-200 Oława, Poland; m.drozd@ergis.eu (M.D.); l.kedzierski@ergis.eu (Ł.K.); 3 Faculty of Production Engineering, Warsaw University of Technology, Narbutta 85, 02-524 Warsaw, Poland; krzysztof.santarek@pw.edu.pl

**Keywords:** cargo security, cargo stability, cargo transport stability level, cargo stability testing, laboratory testing of stability

## Abstract

The paper presents a method and results of experimental testing of the stability of palletized cargo wrapped in stretch film in laboratory conditions and methods and parameterization of its application for proper cargo securing. Reduction of stretch film consumption is also significant for sustainable transport. It will contribute to the minimization of costs on a micro-scale (manufacturers and shippers) and a macro scale—a significant reduction in pollution of the environment and gas emissions by reducing stretch film production. The experiments have been performed following the requirements of EUMOS 40509 and 40511 standards. The proposed method of testing the stability of palletized cargo is based on tests performed on a laboratory test bench using FEF-200 sensors. The results of conducted experiments demonstrated that the selection of a proper stretch film and a cargo wrapping model could significantly reduce the risk of cargo damage through increasing cargo stabilizing forces and, at the same time, reducing stretch film consumption. The developed method can significantly reduce the cost of securing and concurrently assure required cargo security. This directly impacts the safety of all participants in the transport process in supply chains.

## 1. Introduction

Preparation of cargo for transport—proper selection of packaging (shape, resistance to pressure, etc.) and, most importantly, the proper way of forming and securing pallet unit loads (PUL)—is a crucial issue occurring on the side of the manufacturer/shipper, as the first link in the distribution chain [1,2]. Cargo preparation has a huge impact on minimizing cargo damage during transport. Regardless, it is a very often neglected issue. The manufacturer/shipper, in general, is unable to predict the interdependencies between the cargo and packaging components affecting the conditions of transportation performance in supply chains [3,4]. When analysing the rationality of the use of cargo packaging and securing it, only the lowest possible cost of the use thereof is taken into account, without consideration for possible risks of the influence of other factors on the cargo, occurring during transport and storage and losses resulting from damage to products in the packaging as well as the uncertainty of information in the delivery process [5]. Examples of such factors occurring during transport are acceleration/overload forces, vibrations transmitted between the road, the vehicle, and the cargo [6]. Those preparing cargo for shipment also generally overlook proper packaging due to haste or lack of direct responsibility for possible damage.

Losses resulting from cargo damage in supply chains on the Polish or European markets are difficult to estimate due to the lack of data or the lack of access. These losses may go beyond property damage. People lose their lives or get injured because of poor cargo securing for road transport. In 2014, about 1200 people in Europe died in accidents as a result of poor cargo securing [7].

Such a way of packaging and securing cargo should be adopted as the optimum solution where the sum of the costs of packaging and securing and the losses of damaged products is the lowest value while maintaining their functionality. Therefore, economically optimum packaging is considered the one that allows the minimum value of the total costs achieved by the value of the delivery and the permissible incidental losses resulting from damage to the cargo that does not pose a risk to the participants of the transportation process or the environment.

The paper aims to develop and experimentally verify a method for optimum selection of stretch film based on laboratory tests conducted to obtain the required stability of palletized cargo and the method and parameterization of its application, ensuring the proper securing of the cargo. The authors’ proposed approach aims to find the practically verified method of packaging transport unit loads (TUL) using stretch film that will reduce the cost of securing and minimizing cargo damage. The experiments will be conducted following the requirements of EUMOS 40509 and 40511 standards. The method of testing the stability of palletized cargo is based on tests performed on a laboratory test bench using FEF-200 sensors. The results of conducted experiments should also indicate the recommended proper stretch film and a cargo wrapping model that can significantly reduce the risk of cargo damage through increasing cargo stabilizing forces and, at the same time, reducing stretch film consumption. The developed method shall reduce the cost of securing and concurrently assuring required cargo security. This will directly impact increasing the safety of all participants in the transport process in supply chains.

The article is divided into six parts. The first part is an introduction. The second part presents a critical review of the literature on, among others, transport safety concerning cargo stability and legal regulations in this respect, practical tests on cargo stability and experimental methodology, cargo stability theory resulting from the features, and mutual arrangement of packages forming a unit. It also discusses the issue of cargo security in transport. The third part contains a description of the method and assumptions (constraints) of testing the stability of palletized cargo based on tests performed on a laboratory bench using FEF-200 sensors. The fourth part of the article presents the stages of the research in the scope of the tests carried out on the laboratory test bench, together with a proper discussion of the obtained results. Part five presents a discussion of obtained results and conclusions. In the last, sixth part, the authors indicate the recommendations concerning the directions of further research.

## 2. Literature Analysis of the Cargo Safety and Stability

The specific problem studied in our research was the stability of palletized cargo. The research topics on the stability of palletized cargo during transport include several areas relevant to the participants of the transport process and the preparation of the cargo for transport and after transport [8,9]. The developed analytical tool, based on a model mapping the stability of palletized cargo, will allow adjusting the parameters of the stretch film wrapping process to the needs and thus increasing the cargo’s stability. There is little on this topic in the literature.

The literature review in the area of palletized cargo stability can be divided into several areas: transport safety concerning cargo stability, legal regulations on cargo stability, practical research on cargo stability and experimental methodology, theory of cargo stability resulting from the characteristics and mutual arrangement of the packages forming the unit, mechanical properties of the pallet unit resulting from the carrier used, and cargo stability resulting from the packaging materials used. The last area mentioned is particularly relevant to the research presented here.

Cargo transport safety in its broadest sense was discussed from various aspects. Authors of [10] discuss how the use of wrapping materials of various types (which can include stretch films) affects road transport safety, while [11,12] indicated a method to assess the stability of units in transport. Kuskil’din [13] studied the consequences of cargo behaviour in the cargo space during emergency braking. A broader approach to the issue was taken by the author of the paper [14], in which he examined the behaviour of cargo in the context of road transport safety. The selection of means of transport for transport tasks is an important aspect of the research. For example, Ambroziak and Tkaczyk, in their work [15], focus on the selection of means of transport for a transport task, considering the type and way of securing transport units, while in the works [16,17,18], the authors point to the possibility of using evolutionary algorithms to solve complex decision-making problems in this area.

Cargo (including pallet) securing legislation is strongly linked to safety in road transport and other transport modes. How cargo is secured and, indirectly, formed is the subject of Directive 2014/47/EU of the European Parliament and the Council of 3 April 2014 [7]. Methods are being developed to test load rigidity for safety assessment [19,20]. Tkaczyk and Różyk in [21] discuss the responsibility for damage to palletized cargo in transport with reference to the applicable legal acts. The literature also describes in detail ways to secure cargo with stretch film [22].

Cargo stability research is the subject of many studies. It turns out that these are mainly experiment- or simulation-based methods. For example, studies [23,24] comprehensively present methods for testing cargo behaviour during transport, taking into account different transport conditions. Test methods for stability testing are also presented in [25,26], while Danjou and Ostergaard in [27] discuss simulation testing methods of film-secured units. Similar topics are explored in [28,29]. In contrast, Rouillard et al. in [30] introduce simulation as a complement to laboratory tests of cargo stability.

The second element is quite well recognized, as it combines several basic problems: mechanical interactions, cut-and-packing problems, or bin-packing problems. For example, Ratkiewicz and Lewczuk in [31] presented a classification of the aforementioned problems concerning pallet decks. Additionally, they discussed the issue of the influence of the shape of the unit and its selected mechanical properties on the possibility of its storage in a pallet rack. Research on the stability of units resulting from the mutual arrangement of packages was carried out mainly for their computer implementation in the form of palletizing tools. The article [32] presents a three-dimensional bin-packing problem brought down to a linear programming problem. On the other hand, [33,34] discuss the two-dimensional packing problem indicating efficient methods to solve it and the effect of packing patterns on the unit stability. The article [35] represents a wide stream of works focusing on the rational use of cargo space with the proper location of the centre of mass and arrangement of packages forming the cargo. Popiela and Wasiak [6] already focus exclusively on the formation of pallet units by presenting mechanical models allowing for stability estimation. Still, they do not refer to unit protection elements such as stretch film. Kocjan and Holmström [36] discuss the stability of a loaded pallet, but mainly in relation to how it is formed and how much space is used. They mention the possibility of additional measures, but only as an option. The literature on packaging issues is quite extensive and indirectly related to the topic of this paper. Still, it only forms the setting of the issue under study or its boundary conditions.

The issue of wrapping pallet units is also discussed from the point of view of the ergonomics of using stretch film by employees who must apply appropriate force and use the right wrapping technique, as presented in [37]. The wrapping findings presented later in this paper will be the basis for creating work patterns for warehouse workers. The stability of pallet load units is also a reliability factor in supply chains, which indirectly affects the quality of the work performed and the timeliness and costs of logistics (cf. [38]).

The proper selection of stretch films is also crucial from the point of view of the natural environment. Limiting foil consumption will significantly reduce pollution caused by littering the natural environment and reducing gas emissions by reducing foil production. The topics of reducing the carbon footprint are discussed, among others, by Melo, S. and Baptista, P. (using a means of transport that does not emit carbon pollution—cargo bikes) [39]. In many publications, Nocera S. and Cavallaro F. deal extensively with issues related to CO_2_ [40]. On the other hand, Deviatkin et al. also raise environmental carbon pollution issues, including it in pallet production and trade in the difficult and important context [41].

The key issue for the research discussed in this paper is the use of stretch film. For example, the work [42] addressed stretch film behaviour immediately after the filming process itself. The authors show how the film behaves when applied in a certain way and what effect this may have on the cargo, whereas [43] considers the possibility of maintaining cargo stability parameters while minimizing the amount of stretch film used. Similar studies were conducted by Pavlovic A., Kostic S.C., and other authors [44,45,46,47]. Stretch film, when applied, causes pressure on the cargo, which may, in adverse cases, lead to damage to packaging or materials. Research in this area is, therefore, quite popular [48,49,50].

The issues of stability of palletized cargo are discussed by global manufacturers of equipment for such tests [51,52,53,54,55,56,57]), by stretch film manufacturers (including ERGIS Poland [54]), and by associations [22,58,59,60].

Verification of the compliance of the PUL transport stability with the requirements of KSIST FPREN 17321:2021 is determined by the following test methods [61]:

- static test according to EN 12195-1: 2010 (4.2.e) [62],
- dynamic acceleration g test according to Annex A or EUMOS 40509: 2020 (4.2.d) [19],
- dynamic vehicle test according to EN 12642: 2016 (4.2.e) [63].

The KSIST FPREN 17321:2021 standard (intermodal cargo units and commercial vehicles, transport stability of packaging, and minimum requirements and tests), newly developed in 2021, specifies minimum packaging stability requirements and various stability test methods. The PUL transport stability is divided into different levels according to the ability to withstand the respective forces acting on the cargo during transport. Due to different transport stability and various types of PUL, additional cargo securing must be determined in each specific case.

Acceleration values (g) for stability tests have to be in accordance with already existing standards such as EN 12195-1:2010 or related recommendations, e.g., IMO/ILO/12UNICE Code of Practice for Packing of Cargo Transport Units (CTU Code) [60], as described by Tkaczyk in [21,23].

According to the forthcoming standard, the transport stability of the PUL is divided into four levels of transport stability (TSL) based on the horizontal acceleration that the PUL should withstand, according to the values shown in Table 1.

As an association dedicated to improving safety throughout the supply chain, EU-MOS is fully committed to the EU goal of zero road accident fatalities. The EUMOS 40509 and EUMOS 40511 standards are included in Annex III of the European Parliament’s Directive 2014/47/EU on cargo security, which mandates standards to ensure transport security for PUL [58].

Based on the method of dynamic testing of cargo subjected to acceleration g, according to EUMOS 40509:2012, research work was carried out to develop a research method (introduction to optimization) for the selection of stretch film for securing palletized cargo [26].

Research has been conducted around the world on the stability of palletized loads [27]. However, theoretical laboratory tests discussed in the article were carried out in a Polish production company [26] and will allow for verifying test results obtained based on the proposed mathematical model soon. In the world literature, there is no study on the optimization of stretch film consumption as a material ensuring the stability of palletized loads. Verification of the results will allow demonstrating the usefulness of using model tests in practice.

## 3. Safety of Cargo in Transport

**Figure 1:**
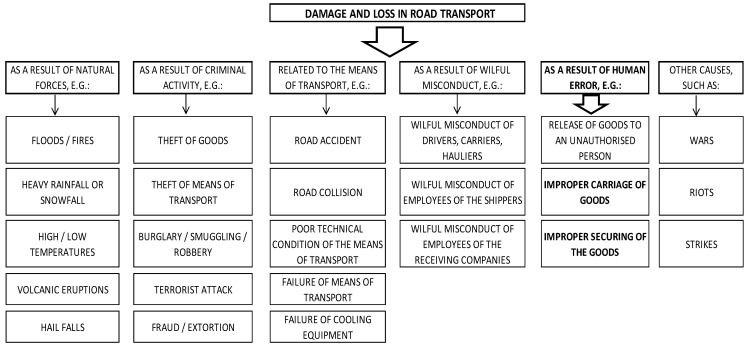
Factors giving rise to losses and damage in road transport.

Many factors can be mentioned that affect damage and loss in road transport (Figure 1). Apart from a few factors beyond human control (forces of nature, natural disasters), most of them arise as a result of intentional (theft, inadequate protection of goods) and unintentional actions (traffic accidents, human error). In this paper, only errors that cause loss and damage, during cargo transportation, due to human error, i.e., damage caused by improper cargo (PUL) preparation, will be considered.

The market for transport services expects transport to be carried out with a guarantee of safety and timely delivery [4,64]. The transportation contract imposes an obligation on the shipper and the carrier to deliver the cargo in its original and undamaged condition [21]. The shipper should strive to pack and prepare the PUL for loading and its further transportation throughout the supply chain [65,66]. The carrier should strive to maintain unchanged usable values of transported cargo at all stages of the transport process. The cargo is exposed to mechanical and climatic, and biological damage. To this end, additional cargo securing on the vehicle should be used if the specifics of the PUL require it. Procedures for applying additional security measures should be applied by carriers (the development of such procedures is one of the objectives of ongoing research work).

**Figure 2:**
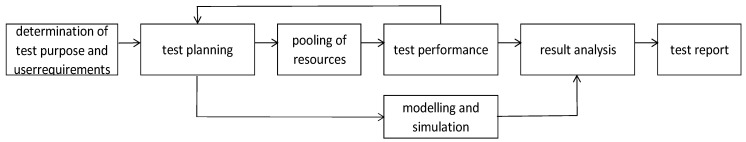
Scheme for the research and testing process.

Mechanical damage is the main cause of damage during transport operations, re-loading, and cargo storage in all transport branches (road, rail, sea, air). However, most damage occurs in road transport, mainly due to the reduction of time devoted to securing the cargo on the vehicle and the desire to reduce the time of transport. The transported cargo is subjected to dynamic loads caused by braking, acceleration, change of travel direction, and unevenness or substantial inclination of the road (Figure 2). Following Directive 2014/47/EU, each cargo shall withstand horizontal acceleration without distortion: 0.8 g in the direction of travel, 0.5 g sideways, and 0.5 g backwards [7].

On the other hand, static loads do not significantly affect cargo damage due to the relatively low stacking height of the cargo on the vehicle if appropriate stacking rules are followed (minimizing the weight of the cargo in the upper layers). The accumulation of static loads and their effect on damage increases with the transport distance, especially for relatively light loads (e.g., cargo transported in cardboard boxes).

Safe cargo is the one that can withstand five-way overloads without damage to the contents and final changes affecting the dimensions of the individual PUL. In practice, this is often very difficult to achieve. Cargo damage is influenced by packaging design features (size, weight, shape) and distribution conditions (distance, shipping relation, handling). Their destructive effect can be inhibited by logistics packaging and cargo securing tools used during transportation.

The most important factor protecting the cargo from damage or destruction is proper packaging and formation of the PUL at the shipper’s site. The damage during transport is often caused by improperly selected or executed packaging (e.g., improperly selected corrugated cardboard in a box) and/or improperly formed PUL (lack of separators, stiffeners, wrapping with stretch film, etc.). In particular, care should be taken to ensure that the collective packages (e.g., cardboard boxes) are placed on the pallet in such a way as to maximize the use of the pallet space also, whether the cargo does not protrude beyond the outline of the pallet, whether the pallet contains the cargo preferably with the same physical properties, homogeneous or similar to the shape of the collective packaging, and whether the shape of the PUL is close to perpendicular. In the case of non-homogeneous PUL cargo, it is recommended that each layer should consist of packages of similar dimensions, and packages of higher weight should be placed at the bottom of the PUL.

## 4. The Method of Testing the Stability of Palletized Cargo

### 4.1. Assumptions of the Method

Testing and research are an integral part of the process of developing new products and technologies (in our case, transport, and packaging of cargo).

The general purpose of testing and research is to confirm (establish) the technical and utility properties of the tested object and, as a result, to reduce the risk accompanying the development of a new product and/or technology. Testing in particular aims at:

- proof of concept for a technical solution,
- ensuring safe operation/use of the technical solution,
- ensuring appropriate ergonomic conditions,
- ensuring that all (defined) customer requirements are met,
- increasing reliability,
- ensuring compliance of project results with the order (contract),
- evaluation of the parameters of the technical solution and the effectiveness of its application,
- investment decision support,
- providing feedback to the designer,
- service quality assessment,
- validation of models used in research—this is usually a separate stage of model building (mathematical, simulation).
- comparison of different variants of design solutions.

Among the many types of research and testing, there are those carried out during research and development works and utility research and tests (operational). Table 2 shows the main differences between the two types of research and tests.

A typical process of conducting research and tests should include establishing the research objective(s), planning the research, conducting the research, often preceded by the construction of a test bench, and then analysing and evaluating the results and drawing conclusions (proposals for further actions), Figure 2.

In the following part of the paper, a description of the method of testing the stability of palletized cargo according to the above scheme will be presented. In the forthcoming next paper, a method for testing palletized cargo using modelling and simulation will be described.

The developed method will enable further research on optimizing the cost of securing the PUL and minimizing cargo damage. This will directly increase the safety of all participants in the transport process.

The research methodology adopted in the article was divided into the following stages:

1. Characteristics of the current situation regarding cargo security in transport. It lists the factors that influence the occurrence of damage and losses. Mechanical damage is characterized. It defines what secure cargo is and what helps protect cargo from damage or destruction during transport. The obligations imposed on the shipper and the carrier by the transport contract concerning cargo security are defined.
2. Formulation of the research problem. The most important factor protecting the cargo from damage or destruction is proper packaging and formation of the PUL at the shipper’s site. The choice of packaging material for the palletized cargo plays an important role here. This poses a significant difficulty for shippers and carriers, resulting in poor cargo packaging and its damage.
3. A study of the stability of palletized cargo. At this stage, two cargo stability tests were performed. The tests differed in two parameters: stretch film with different characteristics/parameters to secure the cargo on the pallet and a different total number of PUL wrappings with stretch film. In each test, two cargo stability tests were performed—verification of the stability of the cargo with set accelerations of 0.4 g and 0.5 g.

### 4.2. Test Stand Description

**Figure 3:**
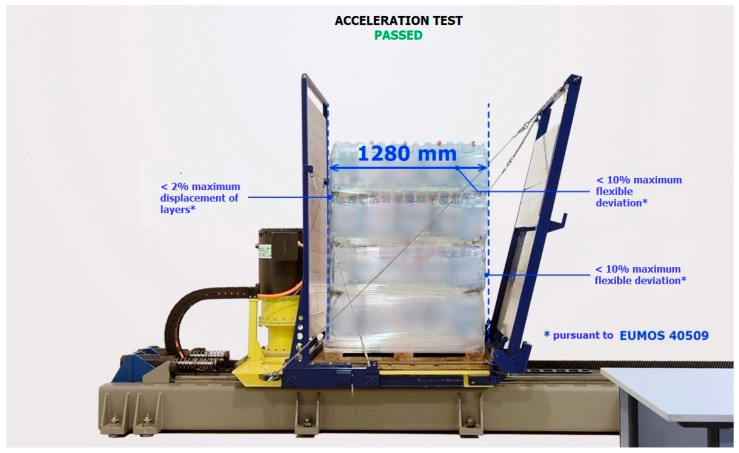
Horizontal stability tester for tests according to EUMOS 40509.

The Horizontal Stability Tester was used to carry out tests following EU-MOS 40509 (Figure 3). The use of a stability tester allows for personalized tests, such as simulating the forces acting on the cargo when a transport vehicle enters a roundabout. The stability tester can add other test procedures on request—not just trapezoidal tests. It can also adapt the machine to the size, weight, and other characteristics required by the samples/cargo to be tested (Figure 3).

The device is equipped with a test platform with the following parameters:

- it is accelerated by an electric motor (according to EUMOS, an acceleration with a duration of at least 300 ms and reached in less than 50 ms),
- it has an inclined front and rear wall of 14°, following the EUMOS standard,
- it has a sliding steel platform with non-slip floor and side guards (to prevent the cargo from falling; the steel basket, inclined on both sides, is designed to perform accelerations and decelerations to analyse the cargo’s behaviour during transport),
- this system has the ability to modify the acceleration value k with an accuracy of two decimal places, which allows for modification of the acceleration time,
- in addition, an optional high-speed camera is available to record the test for later analysis.

**Figure 4:**
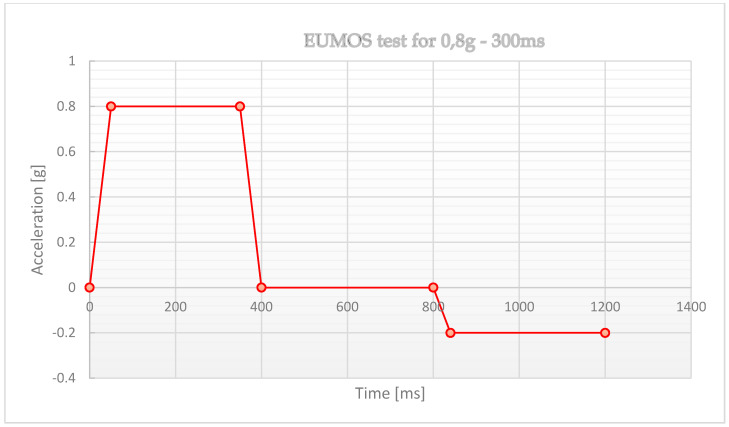
Simulation diagram for acceleration and deceleration of the cargo on the vehicle, according to EUMOS 40509.

The test to be performed to conform to the EUMOS 40509 standard is the stability test, which consists of the following test method:

- place the unit load to be tested on the test platform,
- the platform accelerates until it reaches the programmed acceleration (this acceleration must be reached in less than 50 ms),
- once the target acceleration has been reached, it shall be maintained for at least 300 ms,
- the braking of the platform should be smooth so as not to affect the cargo under test,
- the stiffness level of the cargo is determined by the deformations whose maximum value is defined by the standard (Figure 4).

## 5. Experimental Research

### 5.1. Assumptions for Research on Selection of Film for Cargo Securing

For this paper, load stability tests were carried out at Ergis, a separate research cell, i.e., Ergis Load Stability Academy [57]. The tests aimed to show the impact of packaging on the environment and security in transport.

**Figure 5:**
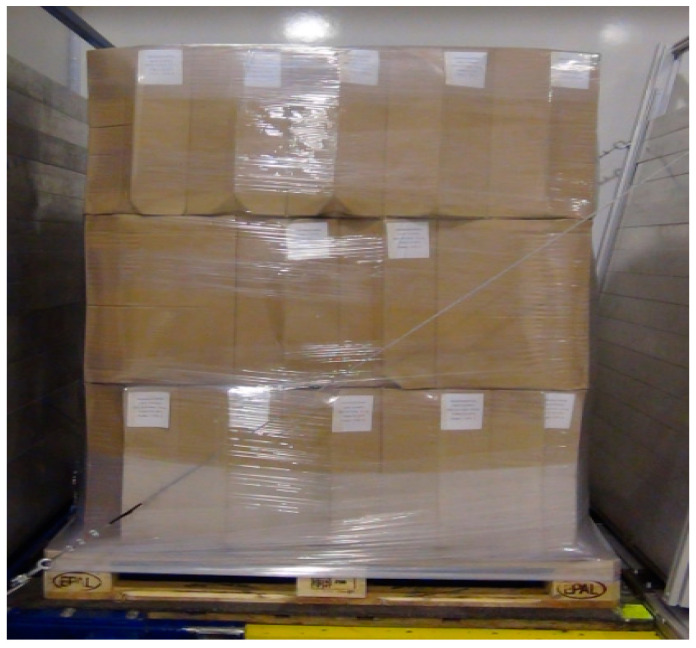
Cargo to be tested—before stability tests are carried out.

For testing purposes, the following assumptions were made:

- the tests aimed to show the impact of the choice of packaging on the environment and security in transport,
- the cargo stability test was limited to testing the effect of securing the PUL with a stretch film,
- the test was divided into the following stages/tests:
  ○ analysis of the PUL wrapping and verification of the technical possibilities of the standard film applied,
  ○ dynamic test—verification of the rigidity of the PUL protected with a standard film,
  ○ the new cargo wrapping application (modification of the wrapping program appropriate for the non-standard film used and the capabilities of the wrapping machine),
  ○ dynamic test—verification of the rigidity of the PUL with a non-standard/dedicated film,
- tests were only carried out with the long side of the pallet parallel to the direction of acceleration,
- a PUL stability at the level of at least 0.5 g was considered a success,
- description of the tested cargo (Figure 5):
  ○ PUL—EPAL pallet with cardboard boxes filled with the manual film was the tested cargo,
  ○ cardboard boxes placed in 3 layers, 11 cardboard boxes in each layer,
  ○ cardboard box dimensions—21 × 33 × 52 mm,
  ○ total cargo height—1.7 m,
  ○ total cargo weight—655 kg.

### 5.2. Wrapping Tests and Verification of Rigidity of Palletized Units Secured with Standard Film

#### 5.2.1. Analysis of Cargo Stabilization Forces for a Standard Film

**Figure 6:**
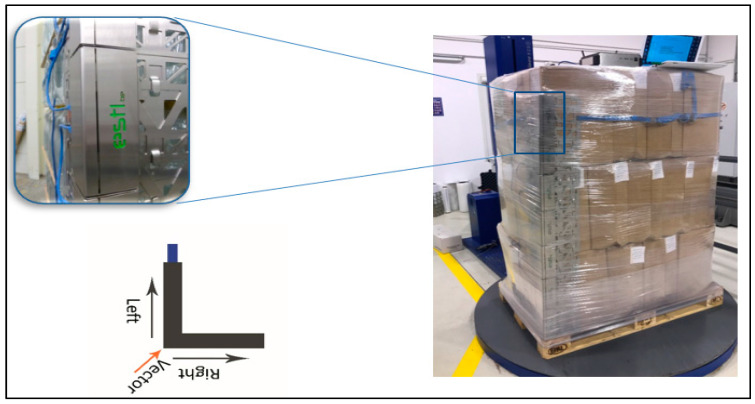
Sensor arrangement FEF-200 on the cargo prepared for testing.

**Figure 7:**
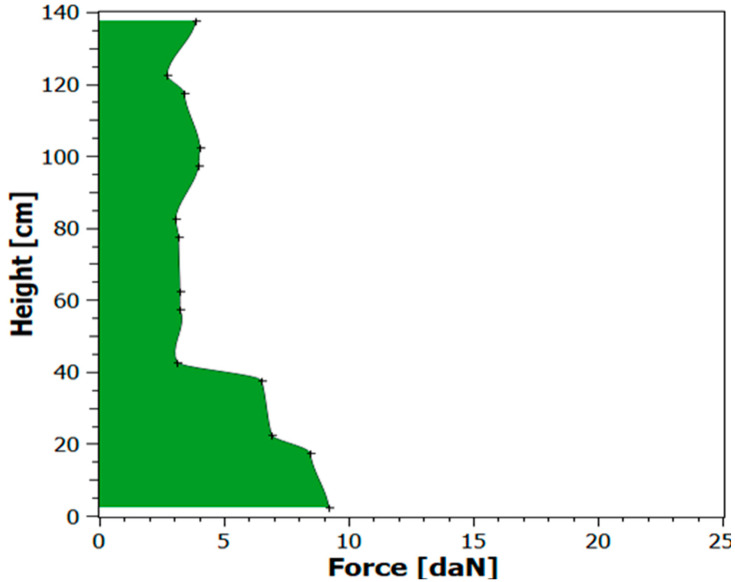
Cargo stabilization forces with the application of the standard film.

The following assumptions were made to conduct the study:

- characteristics of application (cargo wrapping) with standard film (Table 3),
- applied parameters of the programme of cargo wrapping with standard film (Table 4),
- FEF-200 sensors were used as a measuring tool,
- sensors were placed on the cargo before stretch film wrapping to determine the forces exerted by the film on one edge of the cargo,
- the pressure force was measured at 14 points at various cargo heights,
- at each height of the PUL, the vector of the force acting along the long and short side was measured and the resultant force vector was determined (Figure 6),
- the collected data was wirelessly transferred to a computer,
- measured cargo stabilization forces using FEF-200 sensors (Figure 7),
- verification of the technical capabilities of the wrapping machine:
  ○ smooth adjustment of pre-stretch up to 300%
  ○ smooth adjustment of film tension
  ○ ability to change the number of wraps at each stage of wrapping.

#### 5.2.2. Verification of Cargo Stability Level for Non-Standard Film—Acceleration Test

**Figure 8:**
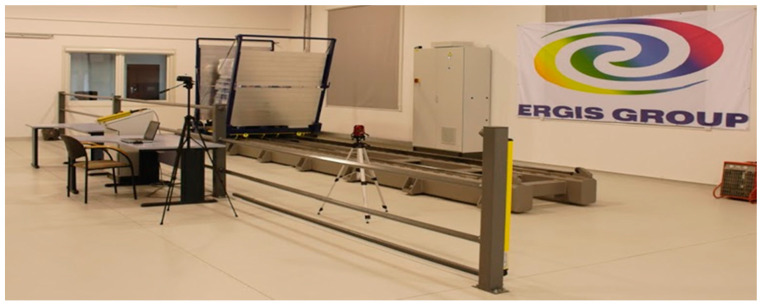
Cargo (PUL) on the acceleration ramp.

**Figure 9:**
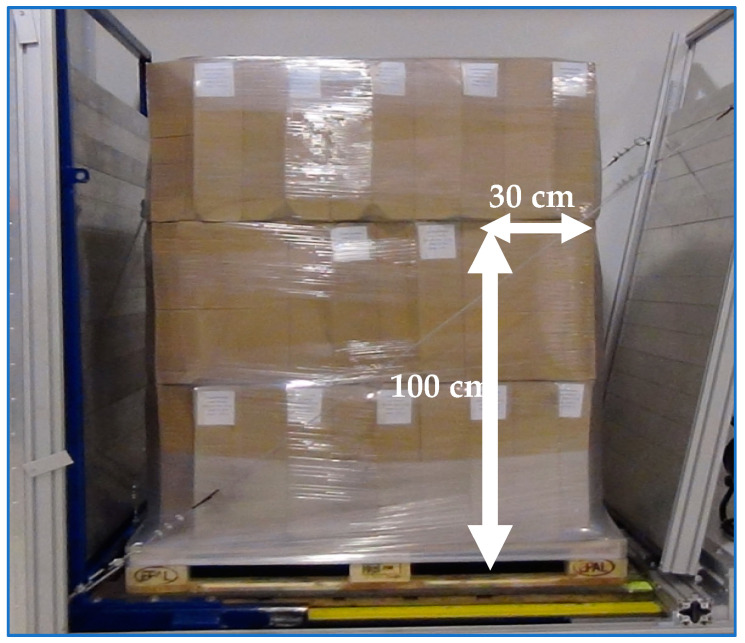
Permissible cargo deviation after stability test.

**Figure 10:**
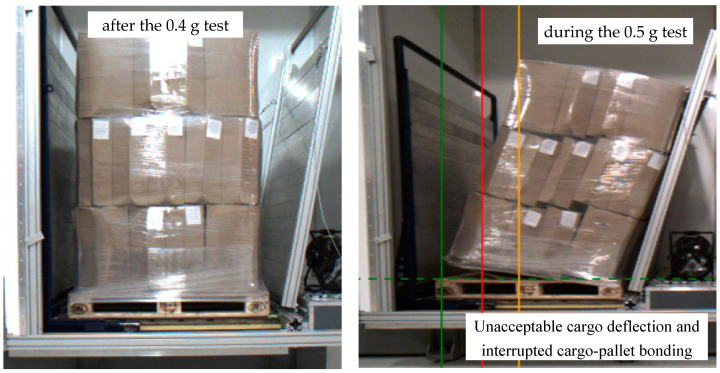
Cargo stability test at specified acceleration of 0.4 g and 0.5 g.

**Figure 11:**
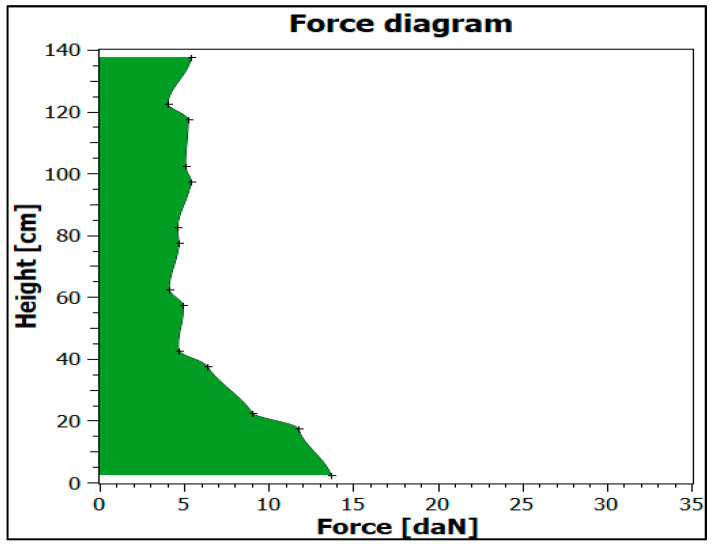
Cargo stabilization forces with the application of the non-standard film.

A full description of the test can be found in EUMOS 40509. Below is a brief description of the test conducted:

- the pallets were loaded onto the mobile truck of the acceleration ramp (Figure 8),
- the ramp has the ability to generate horizontal acceleration in the range of 0–10 m/s² in 0.5 m/s^2^ increments,
- acceleration duration is not less than 300 ms,
- by changing the acceleration value, it is possible to simulate certain traffic situations (sudden acceleration or emergency braking),
- each test is recorded at an accelerated pace for slow motion playback,
- the space for free movement of the cargo during the movement of the ramp is limited (Figure 9),
- the first test—the PUL was subjected to the acceleration of 3 m/s^2^ (0.3 g),
- if the condition of the cargo after the test is satisfactory, the acceleration value is increased by 1 m/s^2^,
- the test shall be considered positive if the deformation of the cargo is not greater than that specified in EUMOS 40509, i.e.,:
  ○ the cargo must not shift more than 5% of its height,
  ○ the cargo must not incline by more than 10% of its height,
  ○ the tested cargo must not be damaged during the test.
- as part of the test performed, further tests were carried out and the maximum acceleration level at which the cargo meets the safety criteria was determined—0.4 g (Figure 10),
- during tests with the set acceleration of 0.5 g, too much deflection occurred and the cargo and pallet bonding was interrupted (Figure 11),
- measured cargo displacements during the test with acceleration of 0.5 g (Table 5).

### 5.3. Wrapping Tests and Verification of Rigidity of Palletized Units Secured with Non-Standard Film

#### 5.3.1. Analysis of Cargo Stabilization Forces for a Non-Standard Film

The following assumptions were made for testing:

- in view of the adjustability of the wrapping machine, the use of multilayer film with a guaranteed stretch of 300% was decided,
- in addition, by analysing the deflection not accepted in the test and the interrupted bonding between the cargo and the pallet, an increase of the number of wrappings at the bottom and in the middle of the cargo was decided,
- new characteristics of application (cargo wrapping) with non-standard film were applied (Table 6),
- applied parameters of the programme of cargo wrapping with non-standard film (Table 7),
- measured cargo stabilization forces using FEF-200 sensors (Figure 11).

#### 5.3.2. Verification of Cargo Stability for Non-Standard Film—Acceleration Test

**Figure 12:**
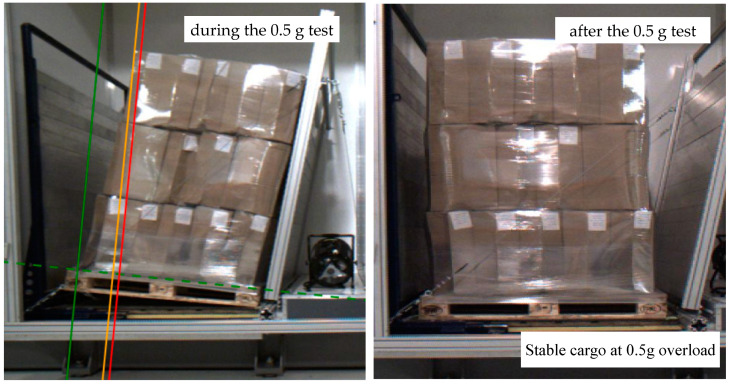
Cargo stability test at the set acceleration of 0.5 g.

A full description of the test can be found in EUMOS 40509. Below is a brief description of the test conducted:

- as part of the test performed, further tests were carried out and the maximum acceleration level at which the cargo meets the safety criteria was determined—0.4 g,
- during the test with the set acceleration of 0.5 g, the cargo was still stable and met the safety criteria (Figure 12),
- measured cargo displacements during the test with the acceleration of 0.5 g (Table 8) were made.

## 6. Discussion of Obtained Results and Conclusions

The tests conducted differed in two parameters: stretch film with different characteristics/parameters to secure the cargo on the pallet and a different total number of PUL wrappings with stretch film.

In each test, two cargo stability tests were performed—the verification of the stability of the cargo with set accelerations of 0.4 g and 0.5 g. More tests were not conducted due to limited funding.

The maximum displacement of the cargo and the maximum deflection of the cargo were evaluated. The test shall be considered positive when the cargo deformation is not greater than that specified in EUMOS 40509, i.e., when the allowable cargo displacement does not exceed 5% of the cargo height and when the maximum cargo deflection does not exceed 10% of the cargo height. Additionally, and obviously, the cargo under test must not be damaged during the test.

The obtained results of cargo stability verification for individual tests are presented in Table 3 and Table 5. Findings of individual tests:

- test 1:
  ○ standard film was used—17 wrappings per PUL, and film weight—448 g/PUL (packaging weight is expressed in grams per PUL),
  ○ the applied standard film was characterized by low parameters of stretch and tension,
  ○ trial 1 (acceleration level 0.4 g)—the cargo met the safety criteria,
  ○ trial 2 (acceleration level 0.5 g)—the cargo did not meet the safety criterion (excessive deflection and interruption of the cargo–pallet bonding occurred),
- test 2:
  ○ non-standard film was used—24 wraps, and film weight—225 g/PUL,
  ○ test 1 (acceleration level 0.4 g)—the cargo met the safety criteria,
  ○ trial 2 (acceleration level 0.5 g)—the cargo was stable, met safety criteria.

**Figure 13:**
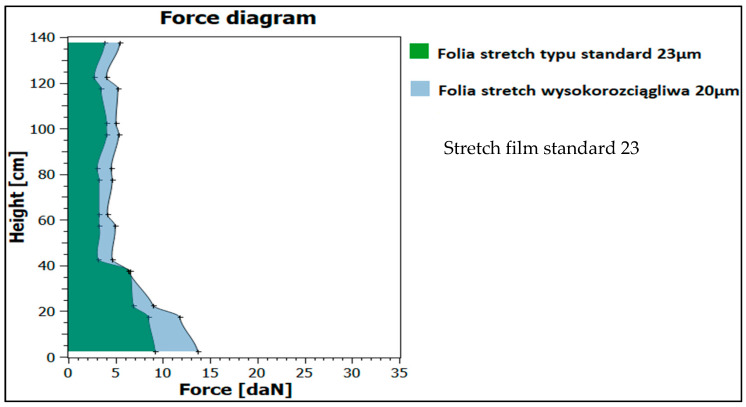
Characteristics of forces for the compared cargo securing films.

To eliminate potential damage to the cargo, when the cargo secured with a standard film did not meet the safety criterion for the acceleration of 0.5 g (test 1, trial 2), a non-standard (dedicated) film was used while changing the wrapping model. An increase in stabilization forces was obtained, which was confirmed by measurements (Figure 13) and an increase in cargo rigidity to the level of 0.5 g (test 2, trial 2).

Application of the new way of packaging cargo will reduce the unit consumption of the stretch film by 50%. On the other hand, it is difficult to determine, due to the business and strategy secrecy of the producer of the non-standard film as well as frequent changes in market prices of film, whether the application of a new method of packaging resulted in a reduction of costs of cargo protection with stretch film. The determination of the level of cost reduction is made on a case-by-case basis for each film user due to the variation in price offered. Furthermore, it can be assumed that increasing the number of wraps from 17 to 24 will not significantly increase the time of this operation, so the costs of wrapping the cargo on a pallet (depreciation of the equipment, cost of employee wages, energy consumption, etc.) for both cases are approximately the same.

It is not easy to unequivocally compare the two tests because the two tests considered different quantitative and qualitative parameters for applying the same cargo using two different kinds of packaging material.

The results of conducted experiments demonstrated that the selection of a proper stretch film and a cargo wrapping model could significantly reduce the risk of cargo damage through increasing cargo stabilizing forces and, at the same time, reducing stretch film consumption. The developed method can significantly reduce the cost of securing and concurrently assuring required cargo security. This directly impacts the safety of all participants in the transport process in supply chains.

## 7. Directions for Further Research

Experiments carried out, discussed in the paper, showed the dependence of the stability of the cargo placed on the pallet on the securing forces determined by the parameters of the film used and the wrapping specification. At the same time, tests have shown that there are films and wrapping methods to meet the EU cargo security requirements.

There is a need to carry out further tests to refine the method of selection of film to secure palletized cargo concerning the cargo properties before stretch film application and to select appropriate settings of packing machines (wrapping machines). The criteria for the selection of the right packaging for palletized cargo have been divided into three groups, taking into account:

- the packaged cargo and its properties before packaging,
- the film and its properties,
- packaging machines (wrapping machines) and their characteristics.

Searching for optimal methods of packing cargo, including the choice of the packaging material, the method of packaging (including the number of layers of packaging material), and taking into account the cost criterion would require a huge number of tests. The duration and cost of such tests are unacceptable.

For these reasons, the next—second—stage of the research will concern the construction of a mathematical model for selecting the optimal (due to costs) method of packing cargo while maintaining the safety requirements contained in the applicable standards and additional requirements and constraints. The results of the experimental research presented in this article will be used to verify the mathematical model.

The development of practical guidelines for logistics operators regarding cargo packaging will be the subject of the third—last—stage of research. Such guidelines must consider many other parameters that were not taken into account during the first (experimental) and second (mathematical modelling) stages of the research. These parameters will apply to, e.g., a wrapping machine. Selecting film without paying detailed attention to the wrapping machine and the cargo itself may not show the totality of the problems faced by customers and stretch film suppliers. Observing the technological progress accompanying the chemistry of plastics, production technology, and constantly increased parameters important from the packaging and logistic point of view, we can see that it should be accompanied by the development of wrapping machines capable of applying the film in a manner maximizing the capacity of the material and calculation methods ensuring correct wrapping from the first time for a new cargo. Such calculations should result from a data collection procedure that collects data on both the film and the wrapping machine, the composition and properties of the cargo to be wrapped, the intended logistics characteristics, and potential risks in the supply chain for that cargo. Such procedures can only emerge from the data collected through both simulation and road tests. Their importance seems to be very high from an economic point of view. Well-packed cargo, safely reaching its recipients, does not pose a risk to the environment. People ensure the optimal use of resources and are a sustainable development source for both businesses and the economy in general.

The method of optimizing the use of stretch film is possible only after considering all criteria for selecting the stretch film (issue under further development) for securing palletized cargo. Developing such a method is a difficult task, and the effort put in will pay off only after the method has been used repeatedly.

## 8. Conclusions

The paper presents a pilot study to confirm the assumptions made to develop a full-scale research program and develop a tool used to optimize stretch film consumption used to secure palletized cargo.

The presented two variants of cargo wrapping using different types of the film show great potential for cargo securing capabilities, while at the same time the direct impact of operating costs (cost of film used, depreciation of the equipment with specific cargo wrapping capabilities, electricity consumption, human labour costs, etc.).

There is, therefore, a need to continue the research work. The result of further re-search work will include:

- development of procedures for stretch film application when wrapping palletized cargo, applied by shippers in supply chains,
- development of procedures for applying additional security measures to improve cargo stability,
- development of a method to optimize the use of stretch film for securing palletized cargo,
- development of a tool for stakeholders (manufacturers and shippers) to assist them in deciding on the level of cargo securing at a cost appropriate to the stability of the cargo,
- the establishment of a certification body to be appointed given the forthcoming new standard EN 17321:2020 (intermodal cargo units and commercial vehicles, transport stability of packaging, minimum requirements and tests), which will set the minimum requirements for packaging stability and various test methods for packaging stability in transport.

Proper selection of stretch film is essential from the point of view of sustainable development of transport. Limitation of film consumption will contribute not only to minimization of costs at the micro-scale (manufacturers and shippers) but also, and perhaps first of all, to the reduction of costs at the macro-scale—a significant reduction of pollution caused not only by littering the environment but also by reduction of gas emission by lowering of film production.

## Figures and Tables

**Table 1:** Transport stability levels (TSL) for PUL (by authors based on [61]).

Transport Stability Level (TSL)	Horizontal Acceleration α
TSL 1	α ≥ 1.0 g
TSL 2	0.8 g ≤ α < 1.0 g
TSL 3	0.5 g ≤ α < 0.8 g
TSL 4	0.18 ^1^ g ≤ α < 0.5 g

^1^ For cargo securing requirements EN 12195-1:2020 below 0.18 g, no TSL marking (not marked).

**Table 2:** Research and tests in the course of R&D work and utility (operational) tests (by authors based on [67]).

Research and Tests in the Course of R&D Work	Utility (Operational) Research and Tests
- tests in a controlled environment, - involvement of qualified users (scientists, designers) - collection of key data - limited number of variables (tested parameters) - focus on the technical parameters (properties) of the tested solution	- tests in a real environment, often different from that envisaged by the designer - users with different background - emphasis on the realism of research, - number of tested variables (parameters) unlimited (theoretically) - focus on the parameters (properties) of the solution

**Table 3:** Parameters of cargo application with standard film.

Material	Stretch Film Standard Type 23 µm
total number of wrappings [wraps/PUL]	17
unit weight of packaging [g/PUL]	448

**Table 4:** Characteristics of cargo application with standard film.

Characteristics	Marking	Value
number of initial bottom rotating wraps	ow^pd^	3
number of initial lower rotating wraps	ow^pn^	1
number of top rotating wraps	ow^g^	2
number of bottom rotating wraps before binding	ow^dw^	0
number of tied rotating wraps	ow^w^	0
starting position of the trolley	pw^s^	0
position of the trolley to slow down during binding	pw^k^	75
stretchability of the film at the start	rf^s^	100
stretchability of the film when wrapping upwards	rf^wg^	100
stretchability of the film at top wrapping	rf^g^	100
stretchability of the film when wrapping downwards	rf^wd^	100
stretchability of the film at bottom wrapping	rf^d^	100
stretchability of the film at binding	rf^w^	100
stretchability of the film at final wrap	rf^k^	100
tensioning force of the film at the start	nn^s^	25
tensioning force of the film when wrapping upwards	nn^ng^	25
tensioning force of the film at top wrapping	nn^g^	25
tensioning force of the film when wrapping downwards	nn^wd^	25
tensioning force of the film at bottom wrapping	nn^d^	25
tensioning force of the film at binding	nn^w^	25
tensioning force of the film at the final wrap	nn^oz^	25
turntable speed	v^s^	10
upward trolley speed	v^wg^	50
downward trolley speed	v^wd^	50

**Table 5:** Cargo displacement parameters during the 0.5 g acceleration test for standard foil.

Maximum Displacements According to EUMOS Standard 40509:	Measurement
maximum cargo displacement—8.5 cm (5% of the cargo height)	top—2.6 cm bottom—0.8 cm
maximum cargo deflection—17.0 cm (10% of the cargo height)	31.4 cm—unacceptable

**Table 6:** Parameters of cargo application with non-standard film.

Material	Multilayer Stretch Film 20 µm
total number of wrappings [wraps/PUL]	24
unit weight of packaging [g/PUL]	225

**Table 7:** Characteristics of cargo application with non-standard film.

Characteristics	Marking	Value
number of initial bottom rotating wraps	ow^pd^	5
number of initial lower rotating wraps	ow^pn^	1
number of top rotating wraps	ow^g^	2
number of bottom rotating wraps before binding	ow^dw^	2
number of tied rotating wraps	ow^w^	0
starting position of the trolley	pw^s^	0
position of the trolley to slow down during binding	pw^k^	75
stretchability of the film at the start	rf^s^	300
stretchability of the film when wrapping upwards	rf^wg^	300
stretchability of the film at top wrapping	rf^g^	300
stretchability of the film when wrapping downwards	rf^wd^	300
stretchability of the film at bottom wrapping	rf^d^	300
stretchability of the film at binding	rf^w^	300
stretchability of the film at final wrap	rf^k^	300
tensioning force of the film at the start	nn^s^	50
tensioning force of the film when wrapping upwards	nn^ng^	50
tensioning force of the film at top wrapping	nn^g^	50
tensioning force of the film when wrapping downwards	nn^wd^	50
tensioning force of the film at bottom wrapping	nn^d^	50
tensioning force of the film at binding	nn^w^	50
tensioning force of the film at the final wrap	nn^oz^	50
turntable speed	v^s^	10
upward trolley speed	v^wg^	40
downward trolley speed	v^wd^	40

**Table 8:** Cargo displacement parameters during the 0.5 g acceleration test for non-standard foil.

**Maximum Displacements According to EUMOS Standard 40509**	**Measurement**
maximum load displacement—8.5 cm (5% of the cargo height)	top—2.2 cm; bottom—1.6 cm
maximum cargo deflection—17.0 cm (10% of the cargo height)	14.3 cm

## Data Availability

Not applicable.
